# Cerebral Disease

**Published:** 1853-04

**Authors:** David Hutchinson


ART. III.—*Cerebral Disease, a sequel of Scarlatina.* By DAVID HUTCHISON, M. D.

B. F., AETAT 21 years old, taken three days ago with sore throat, swelling of the glands and tongue, the throat is ulcerated and has the scarlatinal appearance, tongue much swollen, pulse tense, 100 per minnte, great difficulty of swallowing, much swelled under the chin. Venesect 24 oz.—cath. calomel and jalap—vol. liniment to throat.

Jan. 1st. Tongue fills the mouth, almost impossible to swallow, pulse 90, tense, cannot articulate, made incisions into tongue, bled freely, gave emet. of tart. ant et potass. and ipecac, cath. calomel and jalap.

Jan. 2nd. Articulates well, tongue much reduced in size, can swallow, and is sitting up; his father says that he was delirious during the night, at present rational, pulse 90, contracted and tense, give sulph. magnes. as cathartic, tart. ant et potass. nauseant.

Jan. 3rd. About four o'clock this morning he rose from bed, (he had slept but little for several nights,) put on his coat and socks, and went off; a very heavy rain was falling, he ran two miles to the house of a neighbor, who saw that there was something wrong with him, the neighborhood turned out in search of him, when they found him, and brought him home, apparently in a fit of mental derangement. The pupil of the right eye is double the size of the left, left eye blood-shot, pulse 90, contracted and firm, tongue much improved, deglutition better, articulation good, has a wild, unnatural expression of countenance, venesect 24 oz., cold applications to head, sinopisms to extremeties, sulph. magnesia and tart. emet. sol. till free purgation is produced.

Jan. 4th. Appears rational this morning, pulse 80, softer, expression of countenance more natural, left eye not so much conjested, pupils more equal in size, than on yesterday, appeared better after the action of the cathartic, says that he is weaker, and has lost that quick motion that he had on yesterday, gave dose of calomel and jalap, to be followed by the saline mixture; he speedily recovered, and says that he run off, because he thought that some one was in pursuit of him, to take his life. The pupils of his eyes, in a few days, became equal in size. Several of his brothers, younger than himself, had scarlatina afterwards, attended with much inflammation about the glands of the neck, but no cerebral affection.

